# The Role of Galectin-3 as a Biomarker in the Cardio–Renal–Metabolic Pathology Axis

**DOI:** 10.3390/jcm14176071

**Published:** 2025-08-27

**Authors:** Oana Nicoleta Buliga-Finis, Anca Ouatu, Daniela Maria Tanase, Minerva Codruta Badescu, Nicoleta Dima, Evelina Maria Gosav, Diana Popescu, Ciprian Rezus

**Affiliations:** 1 Department of Internal Medicine, “Grigore T. Popa” University of Medicine and Pharmacy, 700115 Iasi, Romania; oana_finish@yahoo.com (O.N.B.-F.); daniela.tanase@umfiasi.ro (D.M.T.); minerva.badescu@umfiasi.ro (M.C.B.); nicoleta.dima@umfiasi.ro (N.D.); evelina.maria.gosav@umfiasi.ro (E.M.G.); popescu.diana@umfiasi.ro (D.P.); ciprian.rezus@umfiasi.ro (C.R.); 2 IIIrd Internal Medicine Clinic, “Saint Spiridon” County Emergency Clinical Hospital, 700111 Iasi, Romania

**Keywords:** Gal-3, cardio–renal syndrome, heart failure, diabetes, chronic kidney disease, fibrosis

## Abstract

Galectin-3 (Gal-3), a multifunctional protein, plays a pivotal role in a wide range of physiological and pathological processes in the human body. Substantial evidence has linked its overexpression and secretion to the pathogenesis of various conditions, including diabetes mellitus, heart failure, fibrosis, atherosclerosis, and chronic kidney disease. Diabetes mellitus, a persistent metabolic disorder, exerts profound effects on both renal and cardiovascular systems. Contemporary research has investigated a range of various biomarkers aimed at predicting the early onset of renal and cardiac dysfunction in diabetic patients. An early decline in glomerular filtration rate (GFR) may occur even with normal urinary albumin excretion. Given that NT-proBNP concentrations are influenced by GFR, there is a critical need to identify biomarkers capable of detecting early cardio–renal injury in individuals with diabetes. Elevated Gal-3 levels in diabetic patients have been associated with an increased risk of all-cause mortality, cardiovascular disease, and progressive kidney failure and may serve as an indicator of subclinical cardiac and renal dysfunction. Incorporating Gal-3 assessment into clinical practice has the potential to improve diagnostic precision and support personalized management for cardiovascular, renal, and metabolic disorders. This review aims to elucidate the role of Gal-3 as a pivotal biomarker for diagnosis, prognosis, and therapeutic guidance in general in different types of diseases which involve cardio–renal complications.

## 1. Introduction

Galectins constitute a distinct subgroup within the lectin family, characterized by their preferential binding ligands containing β-galactoside structures [1].

The galectin family comprises 15 distinct lectins that are classified into three categories according to the structure of their carbohydrate-recognition-binding domain (CRD). Galectin-1, -2, -5, -7, -10, -13, -14, and -15 comprise the prototype group, which has a single CRD. Based on their structural characteristics, the fifteen distinct galectins that have been found and characterized in humans are divided into three groups: proto-, chimaera-, and tandem-repeat-type. The chimaera group, which only comprises Gal-3, consists of one CRD and one N-terminal domain, while the tandem-repeat group (galectin-4, -6, -8, -9, and -12) features two CRDs connected by a non-conserved sequence [2].

### Gal-3

Gal-3 is a 30 kDa mammalian lectin belonging to the β-galactoside-binding protein family. It consists of three domains: a repeated collagen α-like domain that is susceptible to phosphorylation at certain serines and tyrosines; a short amino terminal domain with 12 amino acids and phosphorylation sites that control its nuclear–cytoplasmic translocation; and a C-terminal that includes a CRD with an NWGR (conserved amino acid motif: N = asparagine (Asn), W = tryptophan (Trp), G = glycine (Gly), R = arginine (Arg)) anti-death motif conserved in the B-cell lymphoma-2 (Bcl-2) protein family [2,3].

Gal-3, first identified in 1984 and subsequently cloned and classified in 1991 as a β-galactoside-binding lectin, is synthesized on cytoplasmic free ribosomes. Lacking a signal sequence, it is not translocated into the endoplasmic reticulum (ER). Gal-3 is widely expressed in a developmentally regulated manner and is present in multiple human tissues and cell types, including kidney, digestive tract, heart, liver, myeloid cells, macrophage, neutrophil, natural killer cells, epithelium of the respiratory tract, and skin.

While Gal-3 is present in the cytoplasm and nucleus of cells, it can also be released into biological fluids and the external environment (e.g., serum and urine). Through its interactions with proteins such as B-cell lymphoma-2 (Bcl-2), which inhibits apoptosis, and guanosine-5′-triphosphate (GTP)-bound K-Ras, as well as its function as a facilitator of p53-dependent apoptosis, Gal-3 plays a significant role in regulating cell survival within the cytoplasm. It facilitates the regulation of gene transcription and mRNA splicing in the nucleus. Gal-3 is necessary for mediating both cell–cell and cell–matrix interactions within the extracellular environment [4].

The LGALS3 gene, located on chromosome 14 at location q21–q22, encodes the human Gal-3 protein. Its promoter region contains multiple regulatory elements, including five predictive Sp1 binding sites (GC boxes), five cAMP-responsive element (CRE) motifs, four AP-1-like and one AP-4-like binding sites, two NF-κB-like sites, a sis-inducible element (SIE), and a consensus basic helix–loop–helix (bHLH) core sequence, suggesting complex transcriptional regulation of LGALS3 expression [5].

Gal-3 is synthesized and subsequently stored in the cytoplasm, where it performs multiple intracellular functions. In response to various stimuli, such as tissue injury or infection, Gal-3 can be either actively secreted by activated cells or passively released from dying or damaged cells. Once present in the extracellular milieu, secreted Gal-3 is proposed to act as a potential damage-associated molecular pattern (DAMP). Additionally, it can function as a pattern-recognition receptor (PRR) and serve as an activator or modulator of innate immunity cells, thereby contributing to the regulation of immune responses.

Gal-3 is involved in numerous physiological and pathological processes such as immune responses, inflammation, angiogenesis, fibrogenesis, cell adhesion, activation, development, differentiation, apoptosis, and cancer progression.

Increased extracellular concentrations of Gal-3 have been associated with a range of human pathologies, including tumor growth, progression, and prognosis; neurodegenerative disorders; and cardiovascular disease [2,4,6].

By recruiting macrophages to sites of tissue injury, Gal-3 plays a pivotal role in the initiation and intensification of the acute inflammatory response. Furthermore, Gal-3 sustains a chronic inflammatory state through the activation of pro-inflammatory signaling pathways. Persistent inflammation, coupled with aberrant tissue repair mechanisms, can ultimately lead to fibrosis, highlighting a critical association between Gal-3 dysregulation and the pathogenesis of various diseases [7].

Gal-3 expression is upregulated in various solid tumor types, and its elevated protein levels are positively correlated with the degree of malignancy. Gal-3 levels were shown to be higher in metastatic tumors compared to primary tumors. Mechanistically, Gal-3 promotes cell survival and proliferation by modulating key oncogenic pathways, including the nuclear factor kappa B (NF-κB) and phosphatidylinositol 3-kinase (PI3K)/protein kinase B (AKT) signaling cascades, thereby contributing to tumor progression and metastasis [8]. Gal-3 stimulates endothelial cells to secrete several pro-inflammatory and pro-angiogenic mediators, including granulocyte-macrophage colony-stimulating factor (GM-CSF), soluble intercellular adhesion molecule-1 (sICAM-1), G-CSF, and IL-6. The interaction of these cytokines with the vascular endothelium upregulates the expression of key endothelial cell surface markers and integrins, such as E-selectin, ICAM-1, vascular cell adhesion molecule-1 (VCAM-1), and αvβ1 integrin, thereby initiating multiple signaling cascades involved in inflammation and metastasis. Elevated serum levels of G-CSF, IL-6, and sICAM1 have been positively correlated with increased serum levels of Gal-3 in patients with metastatic colorectal cancer, suggesting a potential role of Gal-3 in tumor progression and metastatic dissemination [9]. In gastric, thyroid, and osteosarcoma cancers, downregulation of Gal-3 has been shown to effectively suppress tumor cell invasion, migration, proliferation, and metastasis [8] (Table 1). These findings highlight the potential therapeutic value of targeting Gal-3 to limit tumor aggressiveness and impede cancer progression.

## 2. Gal-3 as a Biomarker in Various Diseases

Gal-3 has long been recognized as a biomarker associated with various diseases. However, recent research has underscored its potential as a therapeutic target in the management of numerous fibrotic and inflammatory disorders. Clinically, Gal-3 has already been utilized as a potential clinical biomarker for the early detection of acute heart failure and other forms of cardiac dysfunction [2].

**Figure 1:**
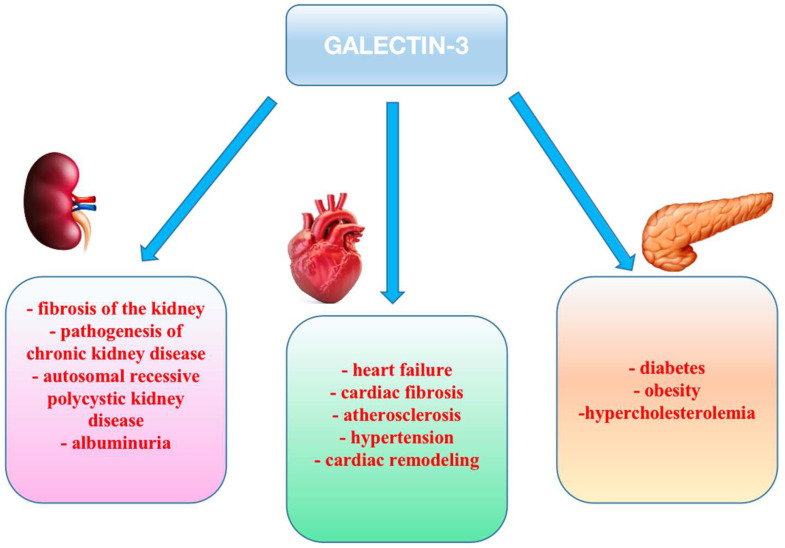
Schematic representation of elevated Gal-3 impact in several organs.

In the general population, circulating Gal-3 concentrations typically range from 10 to 13 ng/mL. An established enzyme-linked immunosorbent assay (ELISA) is commonly employed to measure Gal-3 concentrations in human plasma, providing a reliable and sensitive method for clinical and research applications. In the setting of heart failure (HF), these levels are markedly elevated, potentially reaching concentrations up to 30 ng/mL. The extent of this elevation is closely related with HF severity and is further influenced by comorbid conditions, particularly renal impairment, as Gal-3 levels in HF show a strong correlation with renal function (Figure 1) [10].

Elevated Gal-3 levels have also been observed in individuals with adult congenital heart disease. Recent reports have demonstrated a strong association between Gal-3 concentrations and cardiac function, functional capacity, and the incidence of adverse cardiovascular events in adult congenital heart disease patients [11]. In studies involving adult patients with congenital cardiac disease, Gal-3 levels have also been associated with right ventricle pressure overload [12]. Mohammed et al. [13] demonstrated that Gal-3 serves as a valuable indicator of progressive ventricular remodeling in pediatric patients with congenital heart disease (CHD), showing a positive correlation with pulmonary artery pressure as well as left atrial and ventricular diameters [14]. In adults with congenital cardiac disease, elevated Gal-3 levels have been identified as an indicator of the onset of supraventricular and ventricular tachycardia [15]. Notably, the association between arrhythmia and Gal-3 appears to be independent of other underlying medical conditions. A small-scale study reported that adolescents with ventricular arrhythmias exhibited higher Gal-3 levels compared to their healthy counterparts [16].

Gal-3 has been investigated as a potential biomarker for various human diseases, including thyroid tumors, diabetes, autoimmune diseases, viral infections, and renal disease in addition to heart disease [17]. Additionally, recent studies have identified elevated Gal-3 levels in COVID-19 patients as well as in those with idiopathic pulmonary fibrosis [18].

Extracellular Gal-3 levels may reflect key underlying pathophysiological mechanisms, including inflammation, oxidative stress, and circulating cell infiltration, all of which contribute to the initiation, progression, and rupture of atherosclerotic plaques. Furthermore, in patients with atherothrombosis, circulating Gal-3 concentrations have been associated with clinical outcomes. In a five-year follow-up analysis of peripheral artery disease patients, Gal-3 concentrations were found to be independently and significantly associated with an increased risk of CV mortality (hazard ratio = 2.24, 95% CI: 1.06 to 4.73, *p* < 0.05) [19].

Genetic and epidemiological studies have associated Gal-3 with numerous pathophysiological processes. Evidence from the literature indicates that Gal-3 messenger RNA expression can be detected as early as 30 min following an acute myocardial infarction, may take up to 24 h to become apparent, and can reach peak levels within 14 days [20].

Recent research has demonstrated that anxiety and serum Gal-3 are related in patients with cardiovascular risk factors, regardless of physical disability or serum natriuretic peptide concentrations [21].

Based on current evidence, Gal-3 demonstrates significant diagnostic and prognostic utility in both cardiovascular and non-cardiovascular diseases. Its role in disease progression and outcomes supports its incorporation into clinical risk assessment models and highlights its potential as a promising therapeutic target for the development of novel treatment strategies.

### 2.1. The Role of Gal-3 in Kidney Diseases

In experimental animal studies, Gal-3 has been associated with the development of renal fibrosis. During development, Gal-3 is predominantly expressed in collecting ducts and on the apical membrane of α-intercalated cells. This expression pattern suggests a potential role for Gal-3 in tubular development, possibly mediated through interactions with the extracellular matrix or by facilitating cell–cell adhesion to promote tubulogenesis. Gal-3 expression is seen in main and intercalated cells, proximal tubules, and the ascending thick limb of the adult kidney [22]. Gal-3 was first evaluated by Meijers et al. in a rat model experiment, where it was found to be totally and freely eliminated in urine, exhibiting a clearance of 0.92 mL/min and a distribution volume of 90 mL [10]. The estimated Gal-3 excretion rate in healthy individuals was 3.9 mL/min (2.3 to 6.4), and the fractional excretion of Gal-3 was 3.0% (1.9 to 5.5). Patients receiving hemodialysis can use the dialyzer to filter Gal-3, though at a slower rate than creatinine [10].

Elevated circulating Gal-3 levels have been associated with reduced kidney function and an increased risk of developing chronic kidney disease (CKD). These findings suggest that Gal-3 may serve as a predictive biomarker for kidney injury years before clinical manifestation, thereby enabling early therapeutic intervention targeting and prevention of disease prevention [23].

In a renal-biopsy-based study, plasma Gal-3 concentrations were found to be inversely associated with the estimated glomerular filtration rate (eGFR) and positively correlated with the extent of renal fibrosis [24,25].

In the Framingham Offspring Study, which included 2450 individuals without chronic kidney disease (CKD), elevated Gal-3 levels were associated with a 50% increased risk of CKD events and a more rapid decline in kidney function; however, no association was observed with the incidence of albuminuria. The 10-year trial accounted for factors such as age, gender, diabetes, hypertension, proteinuria, and initial eGFR evaluations. The findings indicate that Gal-3 serves as a predictor of tubulointerstitial fibrosis but not of glomerular injury. The incidence of CKD (eGFR < 60 mL/min/1.73 m^2^) and albuminuria (albumin–creatinine ratio ≥17 mg/g in males, ≥25 mg/g in women) developed in 277 (11.3%) and 194 (10.1%) participants, respectively, while eGFR dropped rapidly (≥3 mL/min/1.73 m^2^/year) in 241 (9.2%) participants [26].

Autosomal recessive polycystic kidney disease (ARPKD), a condition associated with hepatorenal congenital fibrocystic syndromes, may involve Gal-3 as a pathogenic factor. Examining infants with ARPKD both before and after birth revealed that the cystic epithelium was typically positive for an immature form of Gal-3, which was mostly found in the cytoplasm. The observed associations between Gal-3 expression and kidney cyst formation underscore its role in epithelial cell development and proliferation [24].

Analysis of the serum concentrations of Gal-3 in patients awaiting renal transplantation compared with a control group revealed markedly elevated levels prior to transplant. Notably, a significant reduction in serum Gal-3 concentrations was observed three months after the kidney transplant [27].

Recent evidence suggests that targeted anti-Gal-3 therapies have the potential to improve renal function. In patients with stage 3b CKD, a phase II trial comparing Gal-3 inhibitor (GCS-100) medication with placebo revealed a substantial increase in eGFR with GCS-100. Between baseline and the end of therapy, a dose of 1.5 mg/m^2^ contributed to a significant (*p* = 0.045) increase in estimated glomerular filtration rate (eGFR) when compared to a placebo. No statistically significant difference was observed at the level of 30 mg/m^2^ [23,28,29].

Recent studies indicate that serum Gal-3 levels may serve as a predictor of therapeutic response to renal sympathetic denervation. In patients with resistant hypertension, responders to the procedure exhibited significantly higher baseline Gal-3 concentrations compared to non-responders, with no significant changes observed during the follow-up period. These findings suggest that Gal-3 may aid in identifying suitable candidates for renal denervation [30].

In kidney disease, elevated plasma Gal-3 levels are associated with an increased risk of acute renal failure, a higher prevalence of chronic kidney disease, cardiovascular complications, infections events, and multiple causes of mortality in patients with impaired renal function [26].

Experimental and clinical evidence indicates that Gal-3 functions as both a marker and a mediator of tubulointerstitial injury, predicting chronic kidney disease (CKD) progression years before clinical manifestations. Moreover, Gal-3 shows a strong correlation with the extent of renal fibrosis. Collectively, these findings position Gal-3 as a valuable predictive biomarker and a promising therapeutic target in both acute and chronic kidney diseases, with implications for early diagnosis, risk stratification, and development of personalized treatment strategies.

### 2.2. The Role of Gal-3 in Heart Failure

Through its involvement in cardiac fibrosis, inflammation, and ventricular remodeling, Gal-3 plays a pivotal role in the pathogenesis of heart failure [31].

Numerous studies have examined the relationship between Gal-3 and the pathogenesis of atherosclerosis. Gal-3 modulates endothelial dysfunction, inflammation, oxidative stress, and lipid endocytosis processes that contribute to the formation and stability of atherosclerotic plaques. The majority of Gal-3 is localized within the foam cells and macrophages of atherosclerotic plaques, and its increased expression has been positively correlated with the severity of plaque development [32].

Cardiac overexpression of Gal-3 has been shown to induce cardiomyopathy, characterized by myocardial hypertrophy and reduced contractile function [33].

Research has demonstrated that Gal-3 facilitates the recruitment of macrophages to the infarcted myocardium, representing a critical step in the post-myocardial healing process [34,35]. Gal-3 functions as a chemoattractant to draw macrophages to the infarct location. Once there, it binds glycoconjugates on the surface of the macrophages to help them adhere to the endothelium and migrate into the damaged area [36].

Experimental evidence suggests that Gal-3 may mediate, at least in part, the deleterious proliferative effects of aldosterone. In this context, Gal-3 appears to function both as a biomarker and as a causal factor in the development of heart failure with preserved ejection fraction (HFpEF) and heart failure with reduced ejection fraction (HFrEF)/ heart failure with mid-range ejection fraction (HFmrEF) in this setting [37].

In the Diast-CHF observational study, adding Gal-3 to NT-proBNP significantly enhanced the predictive value of the combined model for identifying heart failure with preserved ejection fraction (HFpEF) in 1386 patients at high risk for heart failure or suspected of having heart failure. Although serial measurements of Gal-3 levels over a 6-month period did not improve prognostic value compared to baseline concentrations, high levels of Gal-3 at admission among in-patients with heart failure with reduced ejection fraction (HFrEF) were strongly correlated with higher levels of interleukin-6 and C-reactive protein. Additionally, Gal-3 levels were independently associated with all-cause mortality and heart failure hospitalization [38].

Recent research has demonstrated that cardiomyocytes are also capable of producing galectin. In a cellular model of HFpEF, mechanical stretching of cardiomyocytes led to enhanced Gal-3 expression and significantly increased its the secretion [39].

In patients with heart failure, elevated serum levels of Gal-3 correlate with circulating markers of cardiac extracellular matrix turnover. Longitudinal changes in Gal-3 concentrations may provide a more sensitive and precise predictive biomarker for HF progression. In many patients with HF—particularly those with advanced disease and concomitant renal impairment—Gal-3 expression is markedly elevated. During a 4-month follow-up period in the Valsartan Heart Failure Trial, there was a corresponding increase in the risk of mortality, primary morbid event, and hospitalization for heart failure (2.9, 2.1, and 2.2%, respectively) for every 1 µg/L increase in Gal-3 [40]. Elevated Gal-3 levels are independent predictors of 26-month mortality in patients with chronic heart failure (HF) according to a prospective cohort research study with a 26-month follow-up. A Gal-3 level > 21 ng/mL was linked to higher mortality [41].

The relationship between Gal-3 and other measures of cardiac function remains a subject of ongoing debate. Elevated Gal-3 expression levels, together with increased pulmonary arterial pressure, have been independently associated with higher rates of hospital readmission and all-cause mortality [42].

No significant differences in Gal-3 expression levels have been observed between patients with HFpEF and those with HFrEF. Nevertheless, in HFpEF patients, Gal-3 levels are associated with the severity of diastolic dysfunction and LV stiffness, as well as with adverse clinical outcomes, independent of renal dysfunction and other risk factors. Elevated circulating Gal-3 levels are further linked to structural and functional changes in the LV, suggesting a potential role for Gal-3 in the process of LV remodeling in chronic heart failure [43].

Jiang et al. recently reported that the level of Gal-3 was a risk factor for HFpEF (AUC: 0.763, 95% CI: 0.696–0.821, *p* < 0.01), with a sensitivity of 76.0% and a specificity of 71.9% at the threshold of 15.974 ng/mL [44].

The diagnostic threshold of 10.1 ng/mL was obtained by Kanukurti et al., who recommended combining Gal-3 with NT-proBNP to diagnose HFpEF [45].

Studies have demonstrated that the rate of heart failure increases by 18% for every 1 ng/mL rise in Gal-3 concentration. Furthermore, each standard deviation increase in Gal-3 levels is associated with a 28% higher risk of heart failure [46,47].

Owing to its high sensitivity and specificity, as reported in one study, Gal-3 may serve as an alternative to ultrasonography for the diagnosis of diastolic dysfunction [48].

According to comprehensive biomarker research in heart failure, elevated Gal-3 concentrations in individuals with HFrEF may indicate concurrent right ventricular dysfunction and reduced exercise tolerance [49]. Lu et al. report that the median plasma Gal-3 levels in a group of 166 HFrEF patients was 158.42 pg/mL, and they also show that the diagnostic and prognostic value of this biomarker was determined by the etiology of HF [50]. According to the PARADIGM-HF trial, serum Gal-3 levels at baseline and after eight months did not predict outcomes for individuals with HFrEF [51].

Nonetheless, based on evidence from animal studies—it has been hypothesized that the prognostic value of Gal-3 in HF may be influenced by the underlying disease pathogenesis of HF and the specific therapeutic interventions employed in its management [52].

Even though Gal-3 is a fibrogenic protein essential for normal tissue repair, its prolonged production and secretion within cardiac tissue lead to adverse remodeling, ultimately resulting in progressive fibrosis and the development of heart failure. In numerous animal models, cardiac fibrosis can be decreased by genetic and pharmacological inhibition of Gal-3. Selective inhibitors targeting Gal-3-mediated fibrosis show considerable promise as potential therapeutic agents for the treatment of heart failure [3].

Elevated Gal-3 levels are consistently associated with adverse clinical outcomes, including increased mortality, hospitalization, and disease progression in both HFpEF and HFrEF. When combined with established biomarkers such as NT-proBNP, Gal-3 enhances diagnostic precision and risk stratification. Emerging clinical evidence supports Gal-3 inhibition as a promising therapeutic strategy to mitigate fibrosis and improve cardiac function. However, many studies assessing Gal-3 have relatively short follow-up durations (typically 4–6 months), which may underestimate its long-term prognostic value in heart failure progression. Limited data are available regarding the impact of specific therapies (e.g., RAAS inhibitors, beta-blockers, novel HF drugs) on Gal-3 levels and their influence on its prognostic relevance.

## 3. Gal-3 in Cardio–Renal–Metabolic Syndrome

**Figure 2:**
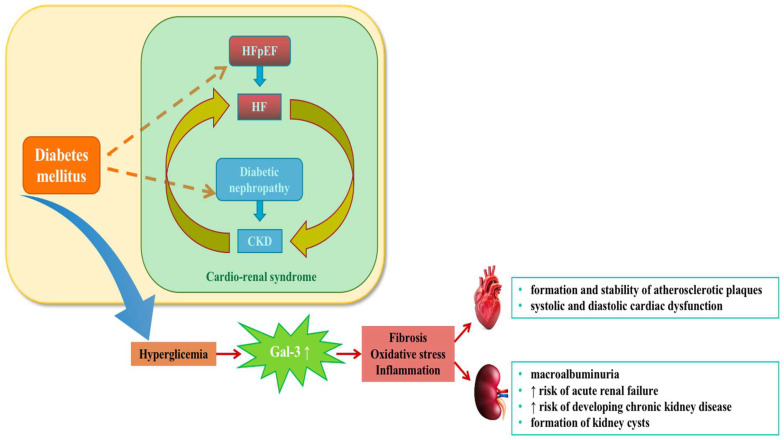
The link between diabetes and cardio–renal syndrome: the role of Gal-3 in cardiac and renal disease; Gal-3, galectina-3, HFpEF, heart failure with preserved ejection fraction; HF, heart failure; CKD, chronic kidney disease; ↑: increase.

Patients with type 2 diabetes (T2D) are at increased risk of developing heart failure (HF) and chronic kidney disease (CKD) as a consequence of metabolic alterations driven by hyperglycemia, hypertension, inflammation, and fibrosis. Fibrosis, a common pathological process in chronic inflammatory conditions, plays an essential role in tissue repair. However, excessive fibrosis can contribute to the progression of CKD and HF, particularly in diabetic patients, culminating in secondary cardio–renal syndrome (CRS) (Figure 2) [53].

Hyperglycemia has been shown to activate metabolic pathways that promote the accumulation of reactive oxygen species (ROS), leading to mitochondrial dysfunction and the upregulation of pro-oxidant enzymes. In addition, hyperglycemia disrupts intraglomerular pressure regulation, resulting in intraglomerular hypertension. This hemodynamic disturbance plays a significant role in the development of cardiovascular disease (CVD) and chronic kidney disease (CKD) in individuals with diabetes [54].

Human studies have also demonstrated that dysregulated glucose homeostasis is associated with elevated Gal-3 concentrations, with significantly higher levels observed in obese and diabetic people [55].

Multiple studies (Yilmaz et al. 2015; Mensah-Brown et al. 2009; Li et al. 2016; Pejnovic et al. 2013; Darrow and Shohet 2015) have reported a strong association between hyperglycemia and Gal-3 levels [56,57,58,59,60,61]. According to Yilmaz et al. (2015) [57] individuals with type 2 diabetes mellitus (T2DM) and prediabetes exhibited significantly higher serum Gal-3 concentrations compared to healthy controls. These levels were positively correlated with fasting plasma glucose (r = 0.787, *p* < 0.01) and 2 h plasma glucose levels (r = 0.833, *p* < 0.01). Furthermore, in their study, Deng Y. et al. reported that Gal-3 expression levels increased with higher glucose concentration in women diagnosed with gestational diabetes mellitus, further supporting the link between Gal-3 upregulation and impaired glucose metabolism [56,57].

### 3.1. Gal-3 Implications in Diabetes

3392 participants in the Dallas Heart Study (DHS) phase 1 (DHS-1) and 3194 participants in the DHS phase 2 had their plasma Gal-3 levels analyzed; 1989 of these patients had paired measurements in both DHS-1 and DHS-2. In addition to having higher weight, waist circumference, and BMI, participants in the higher Gal-3 quartiles were more likely to be older women with hypertension, diabetes mellitus, and hypercholesterolemia. Additionally, lower eGFR and higher levels of hsCRP and IL-18 were linked to higher Gal-3 quartiles.

Recent evidence indicates a strong association between elevated Gal-3 levels and an increased risk of developing diabetes. Patients with type 2 diabetes exhibit significantly higher circulating concentrations of Gal-3 compared to individuals without the disease [57,59,62].

Recent research suggests that Gal-3 may play a significant role in modulating cardiometabolic traits, including insulin resistance, hyperglycemia, and obesity. Gal-3 may influence the risk of cardiovascular disease by modulating key cardiometabolic pathways [63].

Gal-3-deficient mice exhibit accelerated development of diet-induced obesity, hyperglycemia, insulin resistance, and systemic inflammation. In contrast, activation of Gal-3-stimulated receptor-γ, a nuclear receptor involved in adipogenesis, has been shown in murine models to promote adipocyte differentiation [64].

In a study involving approximately 100 participants, Gal-3 levels were positively associated with the presence of diabetes, but contrary to expectations, Gal-3 concentrations in diabetic individuals demonstrated a negative correlation with hemoglobin A1c [63].

Tan et al. [65] demonstrated that in patients with diabetes, Gal-3 concentration was linked to a twofold increase in blood creatinine levels and incident macroalbuminuria [66].

In patients with type 2 diabetes mellitus, Kumar et al. demonstrated highly significant association (*p* value = 0.0001) between elevated serum Gal-3 levels and the presence of both macro- and microalbuminuria. Elevated serum galectin levels and the incidence of non-progressive and progressive retinopathy and neuropathy were also found to be significantly correlated [55].

The precise mechanism underlying the elevated circulating Gal-3 levels observed in diabetic nephropathy remains unclear, as does the exact role in the disease’s pathophysiology. Research by Hodeib et al. demonstrated that mean Gal-3 concentrations were significantly higher in patients with macroalbuminuria compared to those with microalbuminuria and in patients with microalbuminuria compared to those with normoalbuminuria. These findings suggest that circulating Gal-3 levels play a critical role in the onset and progression of diabetic nephropathy [67].

**Figure 3:**
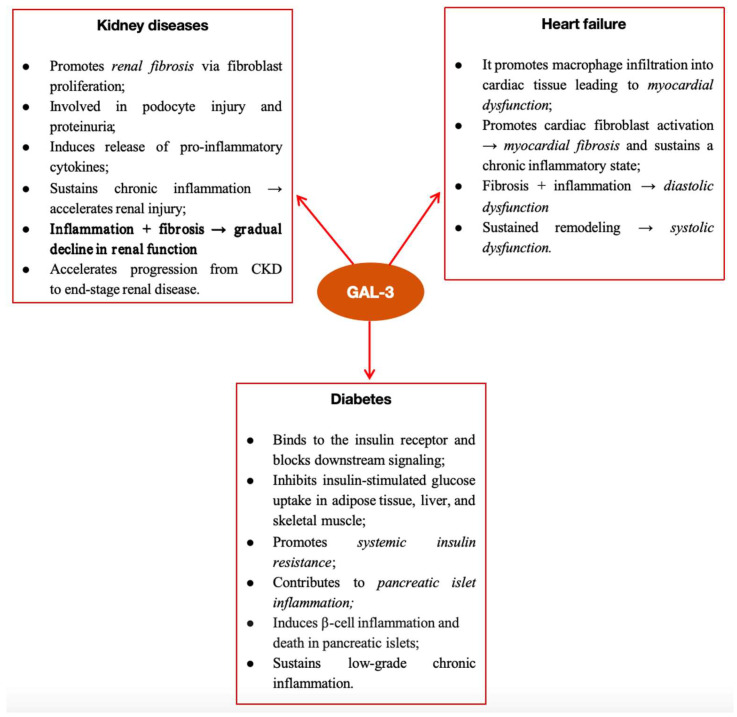
Pathophysiological mechanisms of Gal-3 in different clinical conditions; CKD, chronic kidney disease; TGF-β, transforming growth factor beta.

Insulin secretion, endothelial function, subcutaneous adiposity, and inflammatory indicators were all linked to Gal-3 levels. These findings, supported by additional mechanistic research, suggest that islet-cell inflammation, fibrosis, and eventually β-cell malfunction are the likely mediating factors in the association between Gal-3 and diabetes (Figure 3) [68].

Although microvasculopathy plays a pivotal role in diabetic cardiac remodeling, no biomarker is currently available that can directly monitor the progression of this condition. As a result, the relationship between diabetes mellitus and heart failure is becoming an area of increasing focus. Flores-Ramírez et al. reported that diabetic patients with mildly reduced ejection fraction exhibited elevated Gal-3 levels, which were associated with a reduction in global longitudinal strain [69].

Recent research showed that over a five-year follow-up period, elevated Gal-3 levels strongly predicted the development of both systolic and diastolic cardiac dysfunction in T2DM patients. High levels of Gal-3 were observed in end-stage heart failure, both acute and chronic; they were also linked to the prevalence of diabetes and were even suggested to be a factor in the development of type 2 diabetes mellitus (T2DM) [70].

Given its role in plaque formation and atherosclerosis, Gal-3 levels have also been associated with increased atherosclerotic lesion burden in individuals with type 1 diabetes. As demonstrated by Saeed et al., this increased lesion burden contributes to thromboembolic events in the coronary arteries [71].

Ozturk et al. [72] found a positive correlation between the number of coronary arteries with atherosclerosis and Gal-3 after performing coronary CT scans on 158 patients with T2D [73].

After a mean follow-up of nine years, Tan K.C.B. et al. discovered that even after controlling for baseline eGFR, albuminuria status, and traditional risk factors, Gal-3 was independently linked to the doubling of serum creatinine (HR 1.19; 95% CI 1.14, 1.24, *p* < 0.001) and incident macroalbuminuria (HR 1.20; 95% CI 1.12, 1.30, *p* < 0.001). In type 2 diabetes, serum Gal-3 was found to be independently associated with the progression of renal disease [65].

In summary, current evidence indicates that Gal-3 serves both as a biomarker and as potential mediator of diabetic micro- and macrovascular complications, underscoring its relevance in risk stratification and its potential as a therapeutic target in diabetes management. However existing studies involve heterogeneous populations including individuals with type 1 diabetes, type 2 diabetes, mixed cohorts, and healthy controls, which poses challenges in generalizing findings across all forms of diabetes.

### 3.2. Interferences of Galectin 3 in Cardio–Renal Syndrome

Cardio–renal syndrome encompasses a spectrum of disorders in which acute or chronic dysfunction of the heart can precipitate acute or chronic impairment of the kidneys, and vice versa [74].

CRS is classified into five types:

- CRS type 1: A sudden decline in heart function that results in kidney impairment;
- CRS type 2: Chronic cardiac failure that results in renal impairment;
- CRS type 3: Heart dysfunction brought on by an abrupt deterioration in renal function;
- CRS type 4: Chronic renal dysfunction that results in heart disease;
- CRS type 5: Systemic disorders resulting in concurrent renal and cardiac dysfunction [75].

Given its association with declining renal function, Gal-3 may serve as a valuable predictor of the progression of cardio–renal syndrome. Studies examining the relationship between plasma Gal-3 and renal function in heart failure patients have demonstrated an inverse correlation between Gal-3 concentrations and GFR independent of heart failure status [24].

Patients with elevated Gal-3 levels upon admission were more likely to experience adverse clinical outcomes, such as renal dysfunction, renal tubular damage, and myocardial infarction, among those hospitalized for acute heart failure (AHF). The AKINESIS study demonstrated that elevated Gal-3 levels were associated with the use of inotropes, the need for renal replacement therapy, and in-hospital mortality. Higher Gal-3 concentrations were also linked to an increased composite risk of death or heart failure rehospitalization, as well as death alone, at one-year follow-up. Measuring Gal-3 levels in patients with AHF may help identify individuals at greatest risk for renal complications, myocardial infarctions, and adverse clinical outcomes [76].

In the Framingham Offspring Study, greater Gal-3 levels were associated with a 50% increased risk of incident CKD and with a more rapid decline in renal function among 2450 people without CKD but were not linked to incident albuminuria. The precise relationship between elevated Gal-3 levels and the progression of chronic kidney disease remains unclear. This association may be mediated by independent pathophysiological mechanisms affecting both the kidneys and the heart or, alternatively, by cardiac disease leading secondarily to renal dysfunction [77]. In heart failure patients, reduced fractional clearance of Gal-3 contributes to elevated plasma concentrations of this biomarker. This suggests a complex, reciprocal relationship between Gal-3 levels and renal function in these patients. Elevated Gal-3 levels may both contribute to and result from declining renal function, creating a self-perpetuating cycle of deterioration in the setting of heart failure [78].

Iacoviello et al. identified an independent correlation between renal dysfunction with microalbuminuria in CHF patients and Gal-3 levels. They found that microalbuminuria could be identified with 74% sensitivity and 56% specificity using a Gal-3 cutoff value of 14.2 ng/mL [79].

Recent studies have shown that in patients with reduced left ventricular ejection fraction (LVEF) and symptomatic heart failure, Gal-3 levels may serve as a useful biomarker for predicting the development of cardio–renal syndrome (CRS) [78]. In the study conducted by Ozyildirim et al. [78], the authors investigated the relationship between Gal-3 levels and the development of type 1 cardio–renal syndrome (CRS) in patients with acute heart failure and reduced left ventricular ejection fraction (LVEF). The primary conclusion indicates that patients with CRS had a notably elevated Gal-3 level in comparison to those without the condition (20.7 ± 2.9 ng/mL vs. 17.8 ± 3.1 ng/mL, *p* < 0.001). As an independent determinant of the development of CRS, Gal-3 was found to have a cutoff value of 19.7 ng/mL, with 74% sensitivity and 70% specificity. Additionally, the level of Gal-3 showed predictive power for the development of cardio–renal syndrome (AUC = 0.761, *p* < 0.001) [78].

In HF patients with both preserved and reduced ejection fraction, Gopal et al. [80] examined the relationship between estimated GFR and Gal-3. They found that levels of Gal-3 are strongly correlated with renal function in HF patients [6]. In the study conducted by Ozyildirim et al., higher creatinine (1.5 mg/dL vs. 1.15 mg/dL, *p* = 0.001) and Gal-3 levels (20.7 ± 2.9 ng/mL vs. 17.8 ± 3.1 ng/mL, respectively; *p* < 0.001) were found in HFrEF patients who developed CRS [78].

Additionally, in the Framingham Offspring Study, patients with baseline Gal-3 in the highest quartile and normal renal function (mean eGFR~90 mL/min/1.73 m^2^) had significantly higher odds of developing incident renal dysfunction, which was defined as an eGFR < 60 mL/min/1.73 m^2^ (adjusted OR = 1.47, 95% CI 1.27–1.71, *p* < 0.0001) [81].

In the German Diabetes Mellitus Dialysis (4D) study (1168 dialysis patients with type 2 diabetes mellitus; 4-year follow-up) and the Ludwigshafen Risk and Cardiovascular Health study (2579 patients with coronary angiograms; 10-year follow-up), the association between Gal-3, renal function, and adverse clinical outcomes was evaluated in two large patient cohorts. In patients with impaired renal function, baseline serum Gal-3 levels increased as renal function declined and were independently associated with clinical outcomes such as cardiovascular events, infections, and all-cause mortality. However, these effects were not observed in participants with normal renal function [43].

Studies suggest that elevated serum Gal-3 levels may precede the decline in GFR, potentially reflecting the underlying pathological processes responsible for nephron loss [3].

In patients with chronic heart failure (CHF), higher circulating Gal-3 levels are strongly associated not only with microalbuminuria and reduced glomerular filtration rate (GFR) but also with an increased likelihood of worsening renal function [82] (Table 2).

Serum Gal-3 levels may improve the detection of both cardiac and renal dysfunction, thereby enabling earlier and more accurate diagnosis of cardio–renal syndrome (Table 3).

Elevated Gal-3 levels may both contribute to and result from declining renal function in the context of HF. Ongoing clinical trials will be essential to elucidate the role of Gal-3 in cardio–renal interactions, particularly within systemic conditions such as diabetes, and to assess its potential as a biomarker and therapeutic target in managing combined cardiac and renal impairment.

## 4. Future Perspectives—Anti-Gal-3 Therapy

Gal-3 represents a promising therapeutic target due to its potential for early detection of declining renal function. Additionally, it may be utilized to estimate the risk of CRS progression [93].

Experimental studies in animal models have demonstrated that inhibition of Gal-3 exerts a protective effect against both cardiac and renal injury [94].

In mice with hypertensive nephropathy and heart failure, N-acetyllactosamine, a Gal-3 inhibitor, resulted in reduced proteinuria, improved renal function, and reduced renal injury [95].

In experimental hyperaldosteronism, treatment with modified citrus pectin, a galectin inhibitor, improved cardio–renal dysfunction and prevented aldosterone-induced cardiac and renal fibrosis [96]. Modified citrus pectin inhibited adipose tissue inflammation and decreased the differentiation degree of adipocytes in obese mice, reduced plaque progression in an atherosclerosis mouse model, and prevented isoproterenol-induced LV dysfunction and fibrosis in mice with HF and cardiac-specific hyperaldosteronism [43]. Anti-Gal-3 therapies hold promise for reducing fibrosis and modulating inflammation, offering potential benefits in cardiovascular, renal, and metabolic diseases. Ongoing clinical trials will clarify their therapeutic efficacy and long-term safety.

## 5. Conclusions

Gal-3 has emerged as a multifaceted biomarker and potential therapeutic target in the complex interplay between cardiovascular, renal, and metabolic diseases. Its involvement in key pathological processes—such as fibrosis, inflammation, oxidative stress, and adverse tissue remodeling—underpins its clinical significance in conditions including heart failure, chronic kidney disease, and diabetes mellitus. Elevated serum Gal-3 levels have been linked to an increased risk of all-cause mortality, cardiovascular disease, and progressive kidney failure in individuals with type 2 diabetes. However, the specific role of this biomarker when all three conditions coexist remains to be fully elucidated. Previous studies have underscored the clinical relevance of Gal-3 in patients with heart failure; in heart failure accompanied by acute or chronic kidney disease; in heart failure with diabetes; and in kidney disease coexisting with diabetes.

These findings emphasize the need for further investigation into the utility of Gal-3 as a predictive marker of subclinical renal and cardiac dysfunction in diabetic individuals. Considering the detrimental impact of diabetes mellitus on both the cardiovascular and renal systems, strategies aimed at preventing the onset and progression of these interrelated pathologies are of paramount importance. Experimental evidence supports the potential of Gal-3 inhibition to mitigate organ injury, although robust clinical trials are needed to confirm translational applicability.

The development of Gal-3 inhibitors as therapeutic agents is both innovative and promising; however, further clinical research is required to comprehensively evaluate their potential in the prevention and management of cardio–renal–metabolic diseases.

## Figures and Tables

**Table 1:** The pathogenic roles of Gal-3 in disease development and progression; IL-1β, interleukin 1beta; TNF-α, tumor necrosis factor alpha; IL-6, interleukin 6.

Diseases	Pathogenic Roles of Gal-3
Chronic inflammation	- Promotes recruitment and activation of leukocytes, amplifying the inflammatory response - Stimulates secretion of pro-inflammatory cytokines (IL-1β, TNF-α, IL-6)
Fibrosis	- Maintains a chronic inflammatory state - Activates fibroblasts and myofibroblasts, stimulates production of collagen and proteoglycans
Cancer	- Enhances proliferation, angiogenesis, invasion, and metastasis - Inhibits apoptosis
Cardiovascular diseases	- Involved in post-infarction cardiac remodeling, ventricular dysfunction, and heart failure
Kidney diseases	- Promotes inflammation and fibrotic remodeling in renal tissue

**Table 2:** The utility of Gal-3 in diagnosis, prognosis, and treatment decisions.

Disease	Diagnosis Utility	Prognosis Utility	Treatment Decisions
Acute decompensated heart failure	Diagnostic utility when combined with natriuretic peptides	- Rehospitalization and death are predicted by elevated Gal-3 - Persistent elevation after stabilization predicts long-term adverse outcomes - Predicts the need for renal replacement therapy	- Elevated Gal-3 levels were associated with the use of inotropes - Earlier initiation of antifibrotic or anti-inflammatory therapies
Chronic heart failure	Completes natriuretic peptides	- Strong predictor of disease progression and mortality - Higher circulating Gal-3 levels are strongly associated with microalbuminuria	- Identifies patients who could benefit from antifibrotic therapy
Diabetic nephropathy	Identifies kidney injury early before albuminuria	- Higher Gal-3 concentration correlates with eGFR decline and higher risk of progression to end-stage kidney disease	- Influences the frequency of renal function monitoring

**Table 3:** Main studies with Gal-3 in population with heart failure, chronic kidney disease, and diabetes; ADHF: acute decompensated heart failure; CHF: chronic heart failure; HFpEF: heart failure with preserved ejection fraction; PAD: peripheral artery disease; T2DM: type 2 diabetes mellitus; CVD: cardiovascular disease; CKD: chronic kidney disease.

Author/Study Name	Population	Study Subjects	Results
PRIDE study [83]	ADHF	patients with acute decompensated heart failure	Gal-3 was a prognostic marker for 60-day mortality.
DEAL-HF study [84]	CHF	patients with moderate-to-advanced chronic HF	Gal-3 was an independent predictor of mortality.
Val-HeFT study [40]	CHF	the effect of valsartan in patients with heart failure	Patients with low Gal-3 levels have a reduction in repeat HF hospitalizations.
Care-hf trial [85]	CHF	patients with heart failure, left ventricular dysfunction, and dyssynchrony	Elevated Gal-3 levels were significantly associated with long-term cardiovascular outcomes.
TOPCAT trial [86]	CHF	patients with HFpEF	There are higher Gal-3 levels for this population.
ARIC study [87]	No PAD	9851 participants free of peripheral artery disease	Higher levels of Gal-3 measured were significantly associated with an elevated risk of PAD and critical limb ischemia over 17.4 years of follow-up.
HF-ACTION study [88]	CHF	900 ambulatory patients with HF	Patients with high levels of both NT-proBNP and Gal-3 showed a hazard ratio of 2.19 for hospitalization at 4 years of follow-up compared to patients with low levels of both markers.
Chung, J.O. et al. [66]	T2DM	334 patients with T2DM	Gal-3 concentration was negatively associated with eGFR in patients with T2DM. Moreover, this association was independent of albuminuria status.
Kim, A.J et al. [89]	CKD	352 patients with chronic kidney disease	Gal-3 plasma levels were associated with elevated serum creatinine and urine protein/creatinine ratio and were independently associated with CKD progression.
Rebholz, C.M. et al. [90]	NO CKD and NO CHF	9148 patients with no chronic kidney disease and no chronic heart failure	Gal-3 was higher for low estimated glomerular filtration rate and low urine albumin-to-creatinine ratio and was associated with CKD with an OR of 2.22 95% CI [1.89, 2.60].
Wu, C. et al. [91]	ADFH and CHF	9217 patients with chronic and acute HF	The diagnostic hazard ratios of Gal-3 in predicting mortality in chronic HF patients were 1.13 (95% CI: 1.07–1.21) and 2.17 (95% CI: 1.27–3.08) in AHF patients.
Vora A. et al. [68]	Free of CVD	6586 participants from the Dallas Heart Study	Gal-3 was associated with diabetes prevalence and incidence.
Lin D. et al. [92]	T2DM	405 patients (135 newly diagnosed patients with type 2 diabetes and 270 age- and sex-matched nondiabetic patients)	Gal-3 was increased in T2D.
